# Accessory Mental Foramina in Dry Mandibles: An Observational Study Along with Systematic Review and Meta-Analysis

**DOI:** 10.3390/dj13030094

**Published:** 2025-02-22

**Authors:** Zoi Maria Thomaidi, Charalambos Tsatsarelis, Vasileios Papadopoulos

**Affiliations:** Laboratory of Anatomy, Department of Medicine, Democritus University of Thrace, 67100 Alexandroupolis, Greece; zt221487@students.euc.ac.cy (Z.M.T.); chartsat2@med.duth.gr (C.T.)

**Keywords:** accessory mental foramen, mental foramen, Greek population, prevalence, meta-analysis

## Abstract

**Background/Objectives**: The mental foramen (MF) constitutes a passage for mental nerves and vessels, and it is a crucial anatomical landmark in the body of the mandible. The accessory mental foramen (AMF) is a small, addable foramen proximate to the MF, and it is mainly located posteriorly. The AMF is a rare anatomical variation in human mandibles that must be taken into consideration throughout dental and surgical operations. We aimed to assess the incidence and perform morphological and morphometric analyses of AMFs in the human dry mandibles of the Greek population, in addition to a relevant systematic review and meta-analysis of global data. **Methods**: We studied 114 human adult dry mandibles of unknown gender and age available from the Laboratory of Anatomy, Medical School, Democritus University of Thrace, Greece. We used the search term “accessory mental foramen” in the PubMed, Scopus, and Google Scholar databases to detect all publications of the last 50 years reporting the prevalence and morphology of AMFs in dry mandibles; the search ended on 13 January 2025. Quality assessments were performed using the relevant Joanna Briggs Institute tool. Data were synthesized with the random-effects REML model after Freeman–Tukey double arcsine transformationusing STATA 18. No external funding was received. The PROSPERO CRD is 42025638135. **Results**: According to our data, the MF was present in all observed mandibles, and it was bilateral. Nine AMFs (five right/four left; five round/four oval; six posterior/three anterior to the MF) were found in seven mandibles (five single and two double), and all were unilateral. AMFs presented a mean diameter of 0.96 ± 0.43 mm and mean distances of 4.12 ± 2.15 mm from the MF, 12.68 ± 4.10 mm from the alveolar ridge, and 11.92 ± 1.57 mm from the lower border of the mandible. Furthermore, 27 publications were included in the meta-analysis; the combined AMF prevalence was 6.1% (95% CI: 4.8–7.6%; I^2^ 60%), the combined mean vertical axis was 1.18 ± 0.61 mm, and the combined mean distance from the MF was 3.64 ± 2.29 mm. Bilateral AMFs were detected in 2.1% of AMF cases. An oval shape was described in 37.3% of AMFs. No publication bias was detected. **Conclusions**: AMFs are not considered rare, and they are occasionally bilateral or even multiple in number. Moreover, they demonstrate considerable variation regarding their size, shape, anddistance from the MF, alveolar ridge, and lower border of the mandible. Dental surgeons must be aware of AMFs’ anatomical variations during surgical and anesthetic planning in order to effectively prevent or mitigate the risk of postoperative complications, such as pain, anesthesia, injury, and other adverse outcomes.

## 1. Introduction

The mental foramen (MF) is an oval or circular opening located on the body of the mandible, and it is typically equidistant from its superior and inferior borders. It serves as a passage for the mental nerve, artery, and vein, playing a vital role in innervating the lower face, including the lower teeth, lip, labial mucosa, and gingiva.

The accessory mental foramen (AMF), when present, is identified as a smaller opening located alongside the MF, and it is typically positioned posterior to it. The AMF plays a critical role, particularly during operating procedures, rendering it essential for surgeons to recognize its presence and morphology. Precise knowledge of the variations in the anatomy, shape, size, and position of the MF, along with the presence of the AMF, is invaluable for performing mandibular surgical procedures. These include filling treatments, premolar curettage, root canal therapy, dental implant placement, orthognathic and periapical surgeries, jaw surgeries, and the enucleation of pathologies in the mental region. Given the frequency of dental and maxillofacial procedures involving the mental region, it is imperative for dental practitioners to be well informed about these anatomical details in order to prevent postoperative complications such as lip numbness, which is a significant consequence of improper handling or violation of the AMF [1].

The presence of an AMF is a critical consideration in surgical procedures involving the mental region. These additional foramina house nerves and blood vessels that, if damaged during surgery, can result in complications, such as lower lip numbness or bleeding. Consequently, understanding the location and content of AMFs is essential for planning and executing safe and effective surgical interventions.

Saywer et al. demonstrated that the morphology, position, and incidence of AMFs vary among individuals and across ethnic groups [2]. Additionally, the position of the MF differs between edentulous and dentate subjects [3]. While many studies focus on dry mandibles, others have utilized data from cone-beam computed tomography (CBCT) and panoramic radiographs. Notably, linear measurements from CBCT have been shown to align closely with those obtained from dry mandibles using a digital caliper, the gold-standard method [4]. CBCT measurements of the MF’s size have also been validated for accuracy [5]. However, comprehensive data on AMFs remain limited.

Although numerous observational studies, case series, and case reports have been published, a systematic review and meta-analysis focusing on dry mandibles has yet to be conducted. This study aims to assess the incidence and perform morphological and morphometric analyses of AMFs in human dry mandibles from the Greek population, complemented by a systematic review and meta-analysis of available global data.

## 2. Materials and Methods

The present observational study was conducted on 114 dry adult human mandibles of unknown gender and age, obtained from the osteological collection of the Anatomy Department, Democritus University of Thrace, Greece.

**Figure 1:**
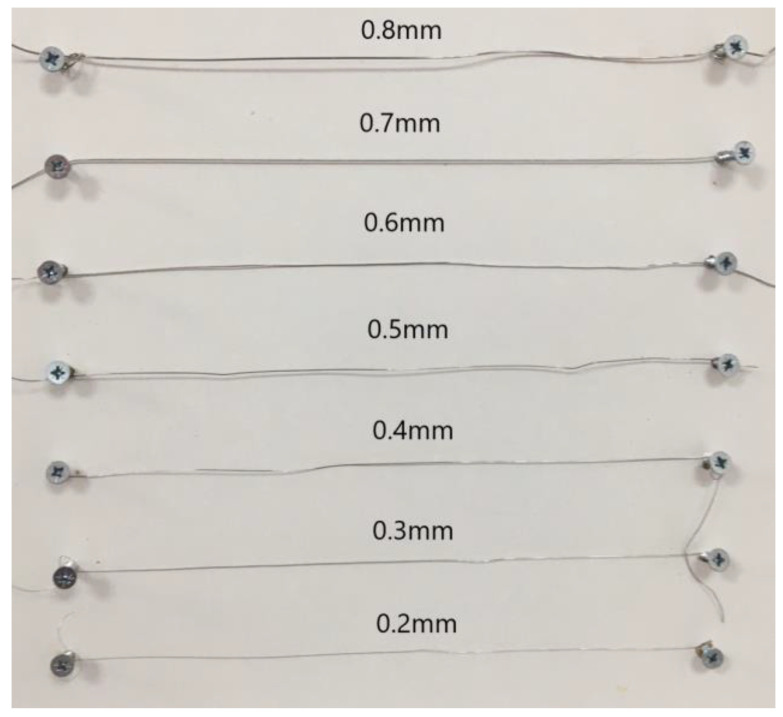
Wires used to additionally determine the diameter of AMFs.

This study’s objectives were to measure the location, shape, incidence, and diameter of AMFs, as well as the distances between AMFs and the MF, alveolar crest, and inferior border in each mandible. For these purposes, we used a digimatic caliper (Mitutoyo Co., Kawasaki, Kanagawa, Japan) with 0.1 mm accuracy. Each distance was measured from the center of each foramen, either the MF or AMF. The location of each AMF was determined using two imaginary lines (horizontal and vertical) intersecting at the MF, dividing the area into four quadrants: anterior superior, posterior superior, anterior inferior, and posterior inferior. The diameter of the AMF was measured using a single stainless-steel wire (UA218893; Zhejiang, China) with a diameter ranging from 0.2 mm to 1.4 mm. For oval-shaped foramina, the horizontal distance was measured using up to two wires with diameters ranging from 0.02 mm to 3 mm (Figure 1).

All measurements were performed independently by two investigators. The inter-rater reliability assessment for categorical variables was assessed using Cohen’s kappa estimates; in the case of continuous variables, the intraclass correlation coefficient (ICC) estimates and their 95% confidence intervals (CIs) based on a mean rating (*n* = 2), absolute agreement, two-way random model were alternatively applied [6]. The means are accompanied by the relevant standard deviation (SD). The two-way random-effects model approach was preferred as we aimed to further generalize our reliability results relative to any raters sharing the characteristics of the selected raters (undergraduate medical or dentistry students) [7]. Based on the 95% confidence interval of the ICC estimate, values less than 0.5, between 0.5 and 0.75, between 0.75 and 0.9, and greater than 0.90 are indicative of poor, moderate, good, and excellent reliability, respectively [7]. The level of statistical significance was set to *p* = 0.05. SPSS software (version 26.0.0.0) was used for statistical analyses.

For the purpose of the present proportion meta-analysis, we implemented a PROSPERO registered protocol (CRD#42025638135), following PRISMA 2020 statement guidelines (Supplementary Table S1) [8]. No amendments to information provided at registration were performed.

We used the search term “accessory mental foramen” in the PubMed, Scopus, and Google Scholar databases to detect all publications reporting the prevalence and morphology of AMFs in dry mandibles; the latter was manually searched for unpublished dissertations and other unpublished studies based on an iterative approach performed until no additional publications could be traced. The Google Scholar search was explicitly extended to every not PubMed-indexed or Scopus-indexed citation identified in the included studies; all identified records were included. The literature search was restricted to papers published over the last 50 years (from 1974) until 13 January 2025. No language limitations were applied. The inclusion and exclusion criteria followed the PICOTS framework (Table 1).

Ethics committee approval was not applicable due to the cadaveric material studied.

Quality assessments were performed using the Joanna Briggs Institute (JBI) Critical Appraisal Checklist for analytical cross-sectional study tools [9].

All data regarding the author, year of publication, origin of population studied, sample size, number of cases presenting AMF, number of cases presenting unilateral AMF, number of cases presenting bilateral AMF, number of total AMFs detected, number of AMFs detected on the right side, number of AMFs detected on the left side, number of single AMFs, number of multiple AMFs, number of round-shaped AMFs, number of oval-shaped AMFs, diameter in mm (if round) or vertical axis in mm (if oval), distance from the MF in mm, distance from the alveolar ridge in mm, and distance from the lower border of the mandible were recorded in a suitable Excel sheet. Quantitative synthesis was performed in cases in which data were available from ≥5 sources. No imputation with respect to missing data was performed.

All steps, including the literature search, record screening, and data extraction, were performed independently by two authors (Z.-M.T. and C.T.). In the case of disputes, a third author (V.P.) was responsible for the final judgment. No automation tools were applied.

Data were synthesized using STATA 18 Statistical Software. Prevalence and proportion data were combined using the random-effects REML model after Freeman–Tuckey double arcsine transformations. The combination of means and standard deviations (SDs) was performed using a freely available online tool: https://www.statstodo.com/CombineMeansSDs.php (accessed on 12 January 2025).

Means and estimates based on the sample size, median, range, and interquartile range were calculated using a freely available online tool: https://www.math.hkbu.edu.hk/~tongt/papers/median2mean.html (accessed on 12 January 2025) [10,11,12].

Heterogeneity was approached using the Q test and I^2^ statistic. A Q test *p*-value of <0.10 was indicative of a statistically significant result. Furthermore, an I^2^ value of ≤25% was indicative of insignificant heterogeneity, 26–50% was indicative of low heterogeneity, 51–75% was indicative of moderate heterogeneity, and >75% was indicative of high heterogeneity [13]. A Galbraith plot was used to assess potential outliers in relation to study-specific effect sizes, their precisions, and the overall effect size. Publication bias was approached via a visual inspection of the symmetry of the relevant funnel plot, aided by the use of Egger’s and Begg’s tests. The trim-and-fill method was implemented to impute “unpublished” studies on either side, thus compensating for any publication bias. Sensitivity analysis was performed using the leave-one-out procedure to investigate the effect of each included study on heterogeneity.

GRADE was used to rate evidence certainty for every endpoint, assessing the risk of bias, imprecision, inconsistency, indirectness, publication bias, and effect size [14]. However, four levels of evidence certainty were used (“very high”, “high”, “low”, and “very low”). In cases in which randomized controlled trials were lacking from included studies, the baseline level is set to “low”. As far as prevalence or proportions are concerned, the effect size is considered small in the case of values of <0.1.

## 3. Results

### 3.1. Primary Data

**Figure 2:**
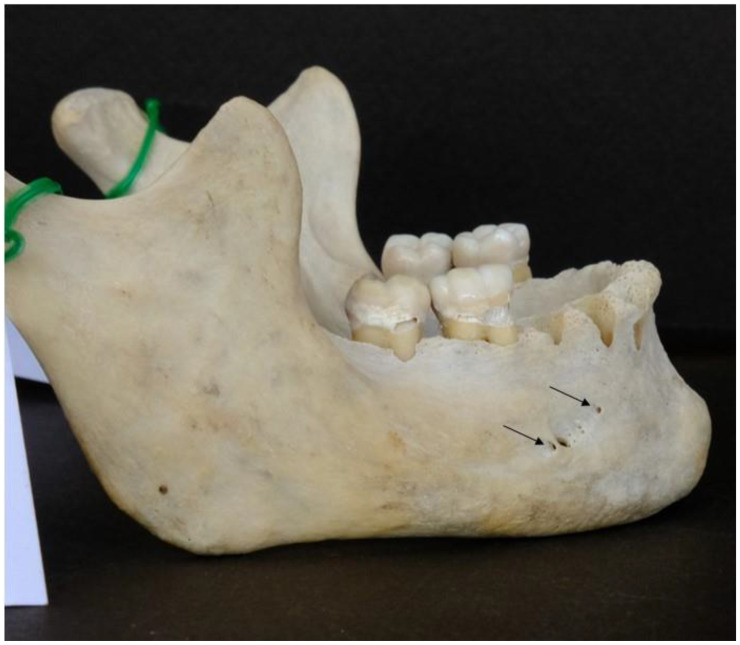
Two small accessory mental foramina located very close to the main mental foramen (top right arrow) and the other one at a far distance from it anterosuperiorly (bottom left arrow)”.

According to our data, the MF was present in all observed mandibles, and it was bilateral. Nine AMFs were found in seven mandibles (five single and two double), and all were unilateral; five AMFs were located at the right hemimandible, and four were located at the left hemimandible (Figure 2).

**Figure 3:**
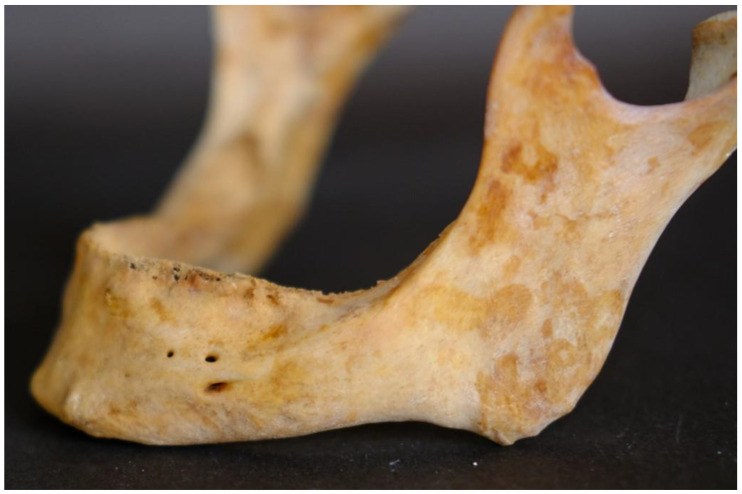
Double accessory mental foramina, round and oval, were found superior and antero-superior to the main mental foramen.

As far as the shape of AMFs is concerned, five were round, and four were oval (Figure 3).

**Figure 4:**
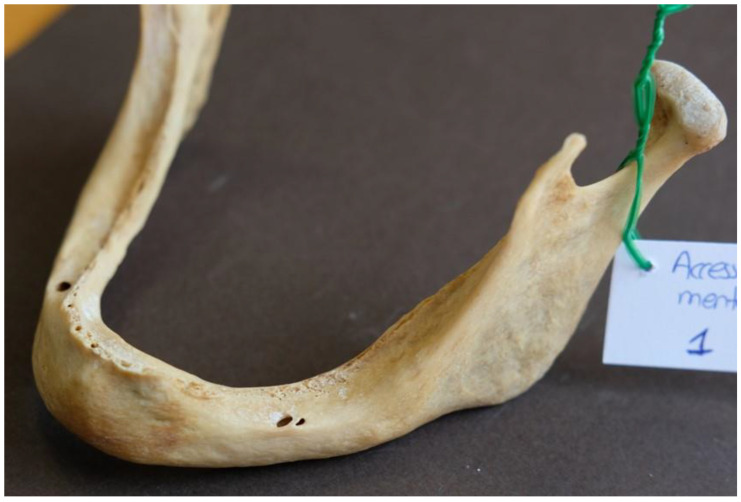
Accessory mental foramen located immediately posterior to the main mental foramen.

As far as the location of the AMFs is concerned, six AMFs were located posterior and three were located anterior to the MF (Figure 4).

**Figure 5:**
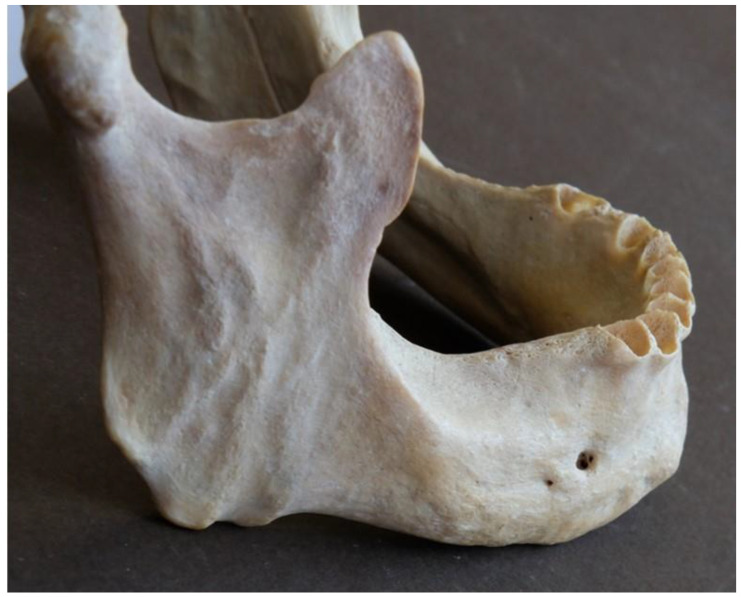
Accessory mental foramen located postero-inferior to the main mental foramen.

Additionally, four AMFs were located inferior to the MF, three superior to it, and two at the same horizontal level as the MF (Figure 5).

**Figure 6:**
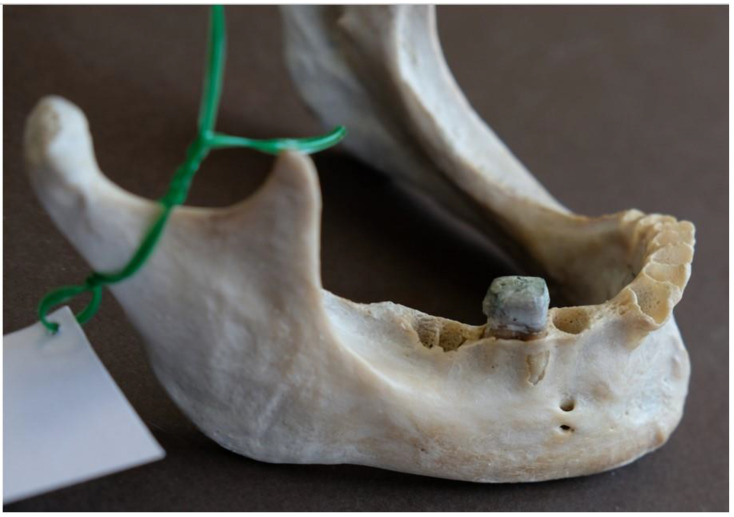
A wide accessory mental foramen located inferiorly to the main mental foramen.

AMFs presented a mean diameter of 0.96 ± 0.43 mm, ranging from 0.40 to 1.70 mm (Figure 6).

**Figure 7:**
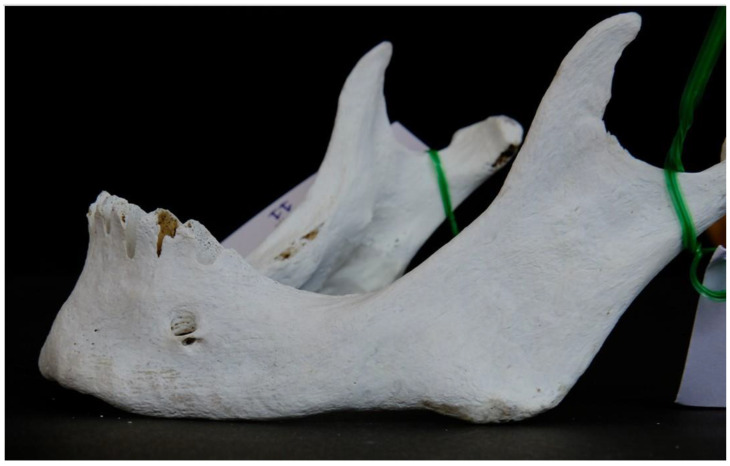
Accessory mental foramen very close to the main mental foramen.

Additionally, AMFs presented a mean distance of 4.12 ± 2.15 mm from the MF, ranging from 3.20 to 17.72 mm (Figure 7).

Of note, AMFs were measured to be located 12.68 ± 4.10 mm from the alveolar ridge and 11.92 ± 1.57 mm from the lower border of the mandible (Table 2).

Lastly, in one mandible, the AMF was located at the same horizontal level as the MF but did not fall within any quadrant (Table 3).

Considerable inter-rater agreement was observed for all measurements (Table 4).

### 3.2. Meta-Analysis Data

For the purpose of the meta-analysis, 252 publications were identified from a search in PubMed and Scopus databases; ten additional publications were retrieved through citation searching (Supplementary Figure S1). No study that appeared to meet the inclusion criteria was excluded for any reason.

After screening, 27 publications were included in the qualitative review and quantitative synthesis, along with data obtained from the present observational study (Table 5 and Table 6).

The quality assessment is presented in detail in Table 7. In particular, eight domains were assessed using respective questions (Q1–Q8) as follows: Q1: “Were the criteria for inclusion in the sample clearly defined?”; Q2: “Were the study subjects and the setting described in detail?”; Q3: “Was the exposure measured in a valid and reliable way?”; Q4: “Were objective, standard criteria used for measurement of the condition?”; Q5: “Were confounding factors identified?”; Q6: “Were strategies to deal with confounding factors stated?”; Q7: “Were the outcomes measured in a valid and reliable way?”; Q8: “Was appropriate statistical analysis used?”. The response options were recorded as “Yes” (Y), “No” (N), “Unclear (U)”, and “Not Applicable” (NA).

**Figure 8:**
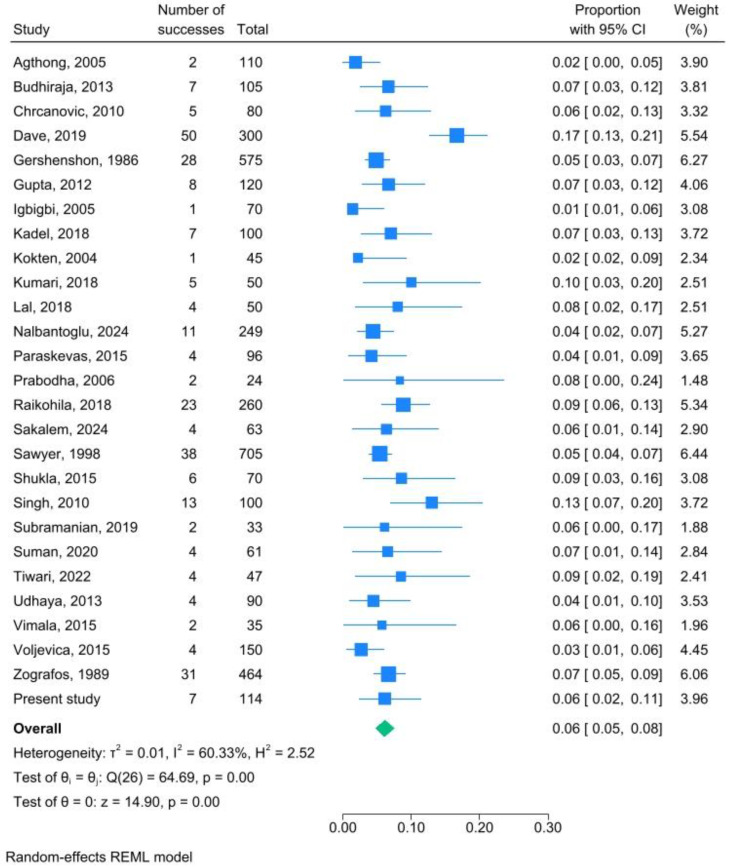
AMF prevalence meta-analysis; the combined prevalence of AMFs in dry mandibles is 6.1% (95% CI: 4.8–7.6%) with I^2^ 60% [2,15,16,17,18,19,20,21,22,23,24,25,26,27,28,29,30,31,32,33,34,35,36,37,38,39,40].

The combined AMF prevalence was 6.1% (95% CI: 4.8–7.6%; I^2^ 60%) (Figure 8).

AMFs were detected in 48.2% (95% CI: 35.2–61.2%; I^2^ 50%) on the right side (Supplementary Figure S2) and in 51.8% (95% CI: 38.8–64.8%; I^2^ 50%) on the left side (Supplementary Figure S3); no difference was documented as the CIs referring to both sides were found to overlap.

The combined mean vertical axis was 1.18 ± 0.61 mm, and the combined mean distance from the MF was 3.64 ± 2.29 mm.

Bilateral AMFs were detected in 2.1% of AMF cases (95% CI: 0.0–7.3%; I^2^ 18%) (Supplementary Figure S4); being a rare anatomic variant, bilateral AMFs are prone to reporting bias.

The oval shape was described in 37.0% of AMFs (95% CI: 5.1–75.8%; I^2^ 83%) (Supplementary Figure S5).

**Figure 9:**
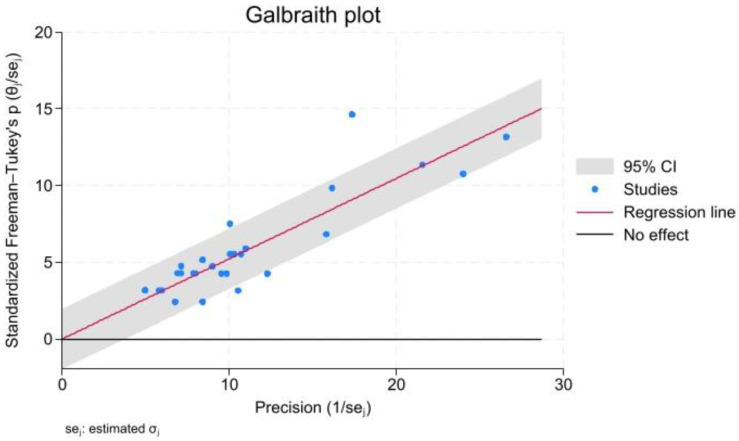
AMF prevalence meta-analysis; Galbraith plot.

All but one study were close to the 95% confidence intervals of the Galbraith plot (Figure 9).

**Figure 10:**
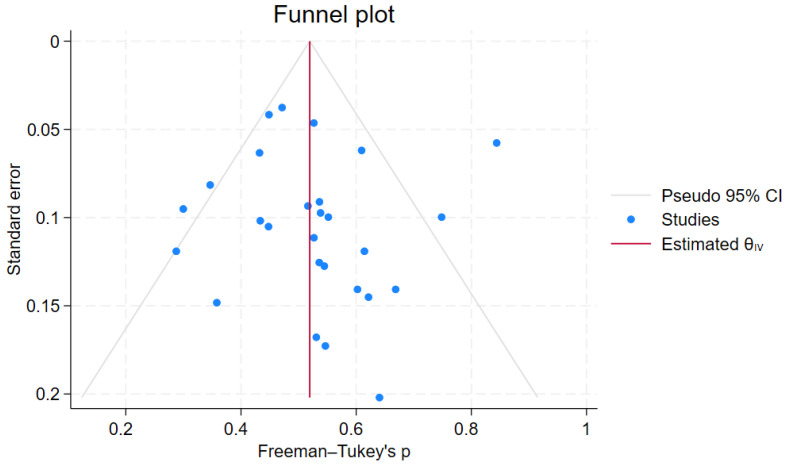
AMF prevalence meta-analysis; funnel plot. Trim-and-fill analysis does not impute any studies on either side. Egger’s *p* = 0.914; Begg’s *p* = 0.234.

No significant publication bias was detected; the funnel plot was symmetrical on visual inspection. Moreover, no additional study was imputed using the trim-and-fill method on either side (Figure 10); both Egger’s and Begg’s tests yielded a non-significant result (*p* = 0.914 and 0.234, respectively).

The leave-one-out sensitivity analysis revealed no critical effect on the combined mean prevalence due to single publications (Supplementary Figure S6).

The GRADE assessment of the certainty of evidence is provided in Table 8. The certainty-of-evidence level is high concerning the endpoints related to the location and shape of AMFs. On the contrary, the overall and bilateral AMF combined prevalence is characterized by low and very low levels of evidence, respectively.

## 4. Discussion

The present observational study investigates the location, shape, and dimensions of AMFs in dry adult human mandibles. The presentation of our data is accompanied by a systematic review and meta-analysis of global data. Notably, this is the first meta-analysis focusing on AMF data obtained exclusively from dry mandibles. In contrast, three previously published meta-analyses primarily relied on CBCT data [41,42,43].

Our data are in keeping with the results of the consequent meta-analysis, thus rendering them credible and representative. Of note, we reported that the combined AMF prevalence is 6.1% (95% CI: 4.8–7.6%) and that there is no difference between the right and left sides. Moreover, AMFs were mostly rounded, while the oval shape was described in 37.0% of cases; the combined mean AMF vertical axis was 1.18 ± 0.61 mm, and the combined mean distance from the MF was 3.64 ± 2.29 mm. Lastly, AMFs were detected bilaterally in 2.1% of cases. The heterogeneity of our results, measured as I^2^, ranged from unimportant to considerable, reaching 83% in the case of AMF shape determination. Both publication bias and the critical effect of any single publication were ruled out as significant sources of heterogeneity. This immediately provides support for the potential contribution of other, as-yet-undetermined parameters, such as gender, origin, and quality of included publications, to the explanation of total heterogeneity.

It is very important to consider accessory mental foramina in surgical procedures involving the mental region of the mandible. These “extra” foramina, which are additional openings in the mandible, house nerves, and blood vessels that could be damaged during surgery, resulting in complications such as lower lip numbness or bleeding. Therefore, understanding their location and content is crucial for surgeons to plan and execute procedures safely and effectively.

The AMF is at risk in many dental and oral surgery procedures carried out in the mental region, such as periapical surgery of the mandibular premolars, genioplasty, bone harvesting from the chin, inferior alveolar nerve repositioning, trauma surgery, and particularly dental implant placement ([44,45,46,47,48,49]). With dental implant procedures being very common, the accessory mental foramen (AMF) represents a key anatomical variation that must be evaluated preoperatively [50,51,52,53]. If the AMF is not identified prior to implant insertion, there is a risk of damaging the accessory mental neurovascular bundle. Such injuries can lead to temporary or permanent numbness, tingling, or pain in the chin and lower lip. Furthermore, the AMF’s location can influence implant placement, potentially requiring the implant to be placed at a different site or angle.

Accessory mental foramina (AMFs) can affect the success of mental nerve blocks, a crucial consideration when administering local anesthesia [54]. If an AMF is located close to the main mental foramen, a single injection of anesthetic solution into the main foramen is often sufficient to anesthetize both. This is because the anesthetic solution can diffuse into the smaller AMF. If an AMF is situated further away from the main foramen, either anteriorly or posteriorly, a separate injection may be necessary to ensure adequate anesthesia. This is because the anesthetic solution from the main foramen injection may not reach the more distant AMF. By understanding the potential variations in AMF location, dental professionals and anesthesiologists can optimize their anesthesia techniques in order to minimize patient discomfort and carry out successful procedures.

In this study, we assessed AMF locations by measuring distances from the AMFs to both the alveolar crest and the inferior border of the mandible. The latter measurement is considered more reliable, as it remains consistent regardless of whether the mandible is dentate or edentulous. In contrast, the distance between the AMF and the alveolar ridge can change due to the resorption of the alveolar ridge with age [3]. However, several studies have demonstrated the superior accuracy and reliability of cone-beam computed tomography (CBCT) when compared to conventional imaging techniques for identifying the accessory mental foramen (AMF) [55,56,57,58,59]. This enhanced visualization makes CBCT an invaluable tool for presurgical planning and execution, enabling clinicians to minimize potential complications and achieve optimal results.

The major strength of this study is its integration of primary data from the well-established osteological collection of the Anatomy Department at Democritus University of Thrace, Greece, with data from a systematic review and meta-analysis. To our knowledge, this is the first study of its kind. However, a certain limitation of this study is that the total heterogeneity, mostly attributable to ethnicity-related variations, could not be further assessed due to the lack of gender-specific information. Moreover, prevalence analyses of rare anatomic variants, such as bilateral AMFs, might be accompanied by considerable reporting bias [60]. Future research incorporating subgroup analyses or meta-regression could explore potential gender-related differences in AMF anatomy.

## 5. Conclusions

AMFs are not considered rare, and they are occasionally bilateral or even multiple in number; moreover, they demonstrate considerable variation regarding size, shape, and distance from the MF, alveolar ridge, and lower border of the mandible. Dental surgeons must be aware of AMFs’ anatomical variations during surgical and anesthetic planning in order to effectively prevent or mitigate the risk of postoperative complications, such as pain, anesthesia, injury, and other adverse outcomes.

## Figures and Tables

**Table 1:** A synopsis of the inclusion criteria using the PICOTS framework.

Parameters	Inclusion Criteria	Exclusion Criteria
(P) Population	Human dry mandibles	Cone-beam computed tomography; panoramic Rx
(I) Indicator (Exposure)	Measurements with digital caliper and wires	
(C) Comparison	Normal/common anatomical condition	
(O) Outcome	AMF prevalence, location, shape, size, and distances from the MF, alveolar ridge, and lower border of the mandible	Cases involving mandible pathology (fracture and tumor)
(T) Time	Papers published over the last 50 years (from 1974)	Previously published duplicates
(S) Setting	Universities (dissection rooms; osteological collections)	

**Table 2:** Characteristics of the accessory mental foramen (AMF) cases of the present study.

**Sample Size**	
Mandibles	114
Sides	228
Mental foramina	228
Accessory mental foramina	9
**AMF cases (per mandible)**	
Absent	107/114 (93.9%)
Present; total	7/114 (6.1%)
Present; unilateral	7/114 (6.1%)
Present; bilateral	0/114 (0%)
**Location**	
Right side	5/9 (55.6%)
Left side	4/9 (44.4%)
Anterior to mental foramen	3/9 (33.3%)
Posterior to mental foramen	6/9 (66.7%)
**Shape**	
Round	5/9 (55.6%)
Oval	4/9 (44.4%)
**Dimensions (mean ± SD); *n* = 9**	
Diameter/vertical axis ^1^ (mm)	0.96 ± 0.43
Horizontal axis (mm)	1.33 ± 0.55
Area (mm^2^)	0.94 ± 0.87
**Dimensions (mean ± SD); *n* = 9**	
From mental foramen (mm)	4.12 ± 2.15
From alveolar ridge (mm)	12.68 ± 4.10
From lower border of mandible (mm)	11.92 ± 1.57

^1^ In the case of oval-shaped AMFs.

**Table 3:** Inter-rater reliability assessment for categorical variables; Cohen’s kappa estimates.

Samples	Side; 1st Rater	Side; 2nd Rater	Position Regarding MF; 1st Rater	Position Regarding MF; 2nd Rater	Shape; 1st Rater	Shape; 2nd Rater
1	left	left	posterior; same horizontal level	posterior; same horizontal level	oval	oval
2	right	right	posterior inferior	posterior inferior	circle	circle
3	right	right	posterior inferior	posterior inferior	oval	oval
4	left	left	posterior inferior	posterior inferior	oval	oval
5A	right	right	posterior inferior	posterior inferior	oval	oval
5B	right	right	anterior superior	anterior superior	circle	circle
6	right	right	posterior inferior	posterior; same horizontal level	circle	circle
7A	left	left	anterior superior	anterior superior	circle	circle
7B	left	left	anterior superior	anterior superior	circle	circle
Cohen’s kappa	1.000		0.805		1.000	
*p*-value	0.008		0.001		0.008	

**Table 4:** Inter-rater reliability assessment for continuous variables; intraclass correlation coefficient (ICC) estimates and their 95% confidence intervals (CIs) based on a mean rating (*n* = 2), absolute agreement, two-way random model.

Parameter	Mean ± SD (Average); *n* = 9	Mean ± SD (1st Rater); *n* = 9	Mean ± SD (2nd Rater); *n* = 9	ICC; 95% CI	ICC *p*-Value
Diameter/vertical axis ^1^ (mm)	0.96 ± 0.43	0.97 ± 0.42	0.94 ± 0.43	0.997; 0.987–0.999	<0.001
Horizontal axis (mm) ^1^	1.33 ± 0.55	1.34 ± 0.56	1.32 ± 0.53	0.997; 0.970–1.000	<0.001
Area (mm^2^)	0.94 ± 0.87	0.96 ± 0.87	0.93 ± 0.86	1.000; 0.996–1.000	<0.001
Distance from mental foramen (mm)	4.12 ± 2.15	4.09 ± 2.15	4.14 ± 2.15	1.000; 0.998–1.000	<0.001
Distance from alveolar ridge (mm)	12.68 ± 4.10	12.66 ± 4.03	12.69 ± 4.17	0.999; 0.998–1.000	<0.001
Distance from lower border of mandible (mm)	11.92 ± 1.57	11.90 ± 1.56	11.93 ± 1.58	0.998; 0.991–1.000	<0.001
Diameter/vertical axis^1^ (mm)	0.96 ± 0.43	0.97 ± 0.42	0.94 ± 0.43	0.997; 0.987–0.999	<0.001

^1^ In cases of oval-shaped AMFs.

**Table 5:** Prevalence meta-analysis; included studies.

Study	Origin	Size	Cases with AMF	Total AMF	Right AMF	Left AMF	Unilateral AMF	Bilateral AMF	Single AMF	Multiple AMFs
Agthong, 2005 [15]	Thailand	110	2	4	2	2	0	2	4	0
Budhiraja, 2013 [16]	India	105	7	7			7	0		
Chrcanovic, 2010 [17]	Brazil	80	5	5			5	0	5	0
Dave, 2019 [18]	India	300	50	51	8	43	49	1		
Gershenshon, 1986 [19]	Israel; India	575	28	39					23	5
Gupta, 2012 [20]	India	120	8	8	5	3	8	0	8	0
Igbigbi, 2005 [21]	Malawi	70	1	1	0	1	1	0	1	0
Kadel, 2018 [22]	Nepal	100	7	7	3	4	7	0		
Kokten, 2004 [23]	Turkey	45	1	1			1	0	1	0
Kumari, 2018 [24]	India	50	5	6	2	4	3	1		
Lal, 2018 [25]	India	50	4	5	4	1	5	0	5	0
Nalbantoglu, 2024 [26]	Turkey	249	11	12			10	1		
Paraskevas, 2015 [27]	Greece	96	4	5	3	2	3	1	5	0
Prabodha, 2006 [28]	Sri Lanka	24	2	2			2	0	2	0
Raikohila, 2018 [29]	India	260	23	27	16	11	19	4	27	0
Sakalem, 2024 [30]	Brazil	63	4	6			2	2		
Sawyer, 1998 [2]	USA	705	42	46	22	20	38	4		
Shukla, 2015 [31]	India	70	6	5	2	3				
Singh, 2010 [32]	India	100	13	13	5	8	13	0	13	0
Subramanian, 2019 [33]	Zambia	33	2	2	1	1	2	0	2	0
Suman, 2020 [34]	India	61	4	5	3	2	3	1		
Tiwari, 2022 [35]	Nepal	47	4	4	0	4	4	0		
Toh, 1992 [36]	Japan			3	2	1	3	0	3	0
Udhaya, 2013 [37]	India	90	4	5	2	3	3	1		
Vimala, 2015 [38]	India	35	2	2	2	0	2	0	2	0
Voljevica, 2015 [39]	Bosnia-Herz.	150	4	4	4	0	4	0	4	0
Zografos, 1989 [40]	Greece	464	31							
Present study	Greece	114	7	9	5	4	7	0	5	2

**Table 6:** Meta-analysis of the shape, size, and distance from the mental foramen; included studies. The combined mean vertical axis is 1.18 ± 0.61 mm, while the combined mean distance from the MF is 3.64 ± 2.29 mm.

Study	Origin	Total AMF	Round	Oval	Vertical Axis (Mean)	Vertical Axis (SD)	Distance from MF (Mean)	Distance from MF (SD)
Paraskevas, 2015 [27]	Greece	5	5	0	1.09	0.15	5.24	3.21
Prabodha, 2006 [28]	Sri Lanka	2	0	2	1.70	0.28		
Raikohila, 2018 [29]	India	27	20	7	1.27	0.77	2.96	2.07
Singh, 2010 [32]	India	13	13	0				
Subramanian, 2019 [33]	Zambia	2	0	2	1.53	0.35		
Tiwari, 2022 [35]	Nepal	4	1	3	1.02	0.03	5.72	0.40
Toh, 1992 [36]	Japan	3			0.84	0.12	2.84	2.13
Present study	Greece	9	5	4	0.96	0.43	4.12	2.15

**Table 7:** Quality assessment of the included studies using the Joanna Briggs Institute (JBI) Critical Appraisal Checklist for analytical cross-sectional studies.

Study	Q1	Q2	Q3	Q4	Q5	Q6	Q7	Q8	Overall Appraisal
Agthong, 2005 [15]	Y	Y	NA	Y	N	N	Y	Y	Include
Budhiraja, 2013 [16]	Y	Y	NA	Y	N	N	Y	NA	Include
Chrcanovic, 2010 [17]	Y	Y	NA	Y	N	N	Y	Y	Include
Dave, 2019 [18]	Y	Y	NA	Y	N	N	Y	NA	Include
Gershenshon, 1986 [19]	Y	Y	NA	Y	N	N	Y	NA	Include
Gupta, 2012 [20]	Y	Y	NA	Y	N	N	Y	NA	Include
Igbigbi, 2005 [21]	Y	Y	NA	Y	N	N	Y	Y	Include
Kadel, 2018 [22]	Y	Y	NA	Y	N	N	Y	NA	Include
Kokten, 2004 [23]	Y	Y	NA	Y	N	N	Y	NA	Include
Kumari, 2018 [24]	Y	Y	NA	Y	N	N	Y	NA	Include
Lal, 2018 [25]	Y	Y	NA	Y	N	N	Y	NA	Include
Nalbantoglu, 2024 [26]	Y	Y	NA	Y	N	N	Y	Y	Include
Paraskevas, 2015 [27]	Y	Y	NA	Y	N	N	Y	NA	Include
Prabodha, 2006 [28]	Y	Y	NA	Y	N	N	Y	NA	Include
Raikohila, 2018 [29]	Y	Y	NA	Y	N	N	Y	NA	Include
Sakalem, 2024 [30]	Y	Y	NA	Y	N	N	Y	Y	Include
Sawyer, 1998 [2]	Y	Y	NA	Y	Y	Y	Y	Y	Include
Shukla, 2015 [31]	Y	Y	NA	Y	N	N	Y	NA	Include
Singh, 2010 [32]	Y	Y	NA	Y	N	N	Y	NA	Include
Subramanian, 2019 [33]	Y	Y	NA	Y	N	N	Y	Y	Include
Suman, 2020 [34]	Y	Y	NA	Y	N	N	Y	NA	Include
Tiwari, 2022 [35]	Y	Y	NA	Y	N	N	Y	Y	Include
Toh, 1992 [36]	Y	Y	NA	Y	N	N	Y	Y	Include
Udhaya, 2013 [37]	Y	Y	NA	Y	N	N	Y	NA	Include
Vimala, 2015 [38]	Y	Y	NA	Y	N	N	Y	NA	Include
Voljevica, 2015 [39]	Y	Y	NA	Y	N	N	Y	Y	Include
Zografos, 1989 [40]	Y	Y	NA	Y	N	N	Y	U	Include
Present study	Y	Y	NA	Y	Y	Y	Y	Y	Include

**Table 8:** GRADE assessment of evidence certainty for every endpoint.

Endpoint	Risk of Bias	Imprecision	Inconsistency	Indirectness	Publication Bias	Effect Size	Evidence Certainty
AMF prevalence (overall)	Not serious	Not serious	Not serious	Not serious	Unlikely	Small	Low
AMF location (right)	Not serious	Not serious	Not serious	Not serious	Unlikely	Large	High
AMF location (left)	Not serious	Not serious	Not serious	Not serious	Unlikely	Large	High
AMF prevalence (bilateral)	Not serious	Serious	Not serious	Not serious	Unlikely	Small	Very Low
AMF shape (oval)	Not serious	Not serious	Not serious	Not serious	Unlikely	Large	High

## Data Availability

The original contributions presented in this study are included in the article/Supplementary Material. Further inquiries can be directed to the corresponding author.

## Supplementary Materials

The following supporting information can be downloaded at: https://www.mdpi.com/article/10.3390/dj13030094/s1, Table S1: PRISMA 2020 Main Checklist; Figure S1: Meta-analysis: PRISMA 2020 flow diagram; Figure S2: Right AMF proportion meta-analysis; the combined prevalence of AMF in dry mandibles is 48.2% (95% CI: 35.2–61.2%) with I^2^ 50%; Figure S3: Left AMF proportion meta-analysis; the combined prevalence of AMF in dry mandibles is 51.8% (95% CI: 38.8–64.8%) with I^2^ 50%; Figure S4: Bilateral AMF proportion meta-analysis; the combined prevalence of AMF in dry mandibles is 2.1% (95% CI: 0.0–7.3%) with I^2^ 18%; Figure S5: Oval-shaped AMF proportion meta-analysis; the combined prevalence of AMF in dry mandibles is 37.3% (95% CI: 5.1–75.8%) with I^2^ 83%; Figure S6: AMF prevalence meta-analysis; sensitivity analysis (leave-one-out analysis).
